# Improved electrochemical performance and solid electrolyte interphase properties of electrolytes based on lithium bis(fluorosulfonyl)imide for high content silicon anodes†

**DOI:** 10.1039/d2ra01233b

**Published:** 2022-04-26

**Authors:** K. Asheim, P. E. Vullum, N. P. Wagner, H. F. Andersen, J. P. Mæhlen, A. M. Svensson

**Affiliations:** Dept. of Mat. Science and Eng., NTNU 7491 Trondheim Norway annmari.svensson@ntnu.no; SINTEF Industry 7465 Trondheim Norway; Institute for Energy Technology 2007 Kjeller Norway

## Abstract

Electrodes containing 60 wt% micron-sized silicon were investigated with electrolytes containing carbonate solvents and either LiPF_6_ or lithium bis(fluorosulfonyl)imide (LiFSI) salt. The electrodes showed improved performance, with respect to capacity, cycling stability, rate performance, electrode resistance and cycle life with the LiFSI salt, attributed to differences in the solid electrolyte interphase (SEI). Through impedance spectroscopy, cross sectional analysis using transmission electron microscopy (TEM) and focused ion beam (FIB) in combination with scanning electron microscopy (SEM), and electrode surface characterization by X-ray photoelectron spectroscopy (XPS), differences in electrode morphological changes, SEI composition and local distribution of SEI components were investigated. The SEI formed with LiFSI has a thin, inner, primarily inorganic layer, and an outer layer dominated by organic components. This SEI appeared more homogeneous and stable, more flexible and with a lower resistivity than the SEI formed in LiPF_6_ electrolyte. The SEI formed in the LiPF_6_ electrolyte appears to be less passivating and less flexible, with a higher resistance, and with higher capacitance values, indicative of a higher interfacial surface area. Cycling in LiPF_6_ electrolyte also resulted in incomplete lithiation of silicon particles, attributed to the inhomogeneous SEI formed. In contrast to LiFSI, where LiF was present in small grains in-between the silicon particles, clusters of LiF were observed around the carbon black for the LiPF_6_ electrolyte.

## Abstract 2

Lithiation of silicon in an LiFSI electrolyte results in a bilayer SEI, with an inner, inorganic layer, and an outer, organic. This SEI is more conductive, flexible and homogeneous compared to the SEI formed in an LiPF_6_ electrolyte.

## Graphical abstract

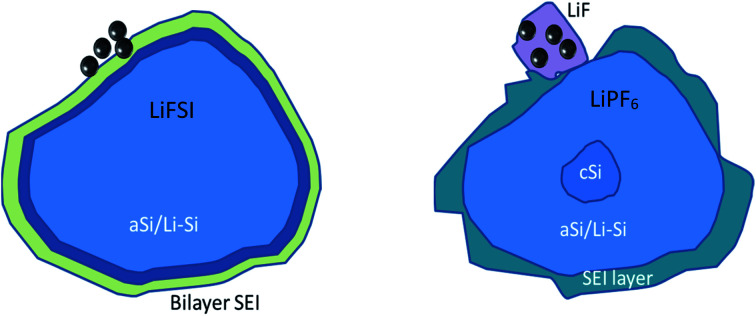

## 1 Introduction

Moving towards a greener future, there is a continuously increasing need for LIBs with higher energy density. Silicon has a theoretical capacity about 10 times higher than graphite, but the main challenge is the large volume expansion (around 300%) associated with the high capacity.^1,2^ This places large strains on the silicon particles themselves and especially on the solid electrolyte interphase (SEI) layer, resulting in cracking of both, and potentially more SEI formation which again consumes lithium. Thus, strengthening or increasing the ductility of the SEI is one of the important measures to enable the use of high fractions of silicon in commercial cells.

For conventional LiPF_6_-based electrolytes, it is generally agreed that the SEI formed in these electrolytes mainly consists of the solvent reduction products lithium ethylene dicarbonate (LEDC), ROCO_2_Li, ROLi, and reduction products from salt and impurities such as Li_*x*_PF_*y*_, Li_*x*_PF_*y*_O_*z*_, LiF, Li_2_O, and Li_2_CO_3_.^3–5^ The structure of the SEI has been described as both “mosaic”, and as a bilayer structure with an inner, “dense”, inorganic layer, and an outer, “soft”, organic layer. The inner layer is typically composed of LiOH, Li_2_O and LiF.^6,7^ The inorganic component Li_2_CO_3_ is an exception, as it has been found to be more widely distributed in the SEI.^8^ For silicon electrodes, in addition to the SEI bilayer model, the inorganic layer can again be divided into two parts.^9^ An outer layer formed from electrolyte decomposition containing mostly LiF and an inner layer formed from lithiation of the native oxide layer containing mostly Li_*x*_SiO_*y*_, Li_*x*_Si, and Li_2_O, in addition to SiO_2_.^9,10^ After just exposure to LiPF_6_ electrolyte, the fluorinated species SiO_*x*_F_*y*_ is also found in this bottom layer.^11^ For silicon electrodes, the SEI tends to be thick and non-uniform, due to the volume changes of the Si, causing cracks and delamination of the SEI, leading to repeated formation and growth, and hence continuous consumption of the electrolyte. The resulting SEI is therefore non-uniform,^12–14^ and with poor Li-ion conductivity.^15–17^ Eventually the dynamic growth leads to clogged electrode pores which impede electrolyte access, increases polarization and hinders Si lithiation.^1,18,19^ Electrolyte additives such as fluoroethylene carbonate (FEC) and vinylene carbonate (VC) are commonly used in combination with silicon electrodes to build a strong and more stable SEI, resulting in higher coulombic efficiency and longer cycle life.^20–27^ These additives decompose at higher potentials, forming denser, more even SEI with higher amounts of LiF compared to ethylene carbonate/diethyl carbonate (EC/DEC).^28–31^ It should be noted that regarding the performance improvements observed upon additions of FEC and VC, several studies have suggested that cross-linking of the polycarbonates is the main reason for a more stable SEI upon additions of FEC and VC,^20,22,26,30,32^ such that the improvements are not directly correlated to the presence of LiF. A recent review of electrolyte additives is provided in ref. 33.

One major disadvantage of LiPF_6_ is the fact that HF is inevitably formed in the presence of trace amounts of H_2_O. In combination with Si electrodes, the presence of HF will lead to fluorination of the native oxide layer, thus forming *i.e.* SiO_*x*_F_*y*_ species, and it will etch the advantageous Li_2_O component of the SEI.^11^ An alternative salt, less prone to hydrolysis, is LiFSI, which has been found to improve performance of nano-silicon electrodes^34^ and graphite electrodes,^35,36^ compared to electrolytes with LiPF_6_. The improvements of LiFSI based electrolyte has been attributed to the lack of fluorination and etching of the Li_2_O formed.^34^ Other favourable features with LiFSI compared to LiPF_6_ is the lower overpotential with graphite electrodes, with lithiation plateau starting at a higher voltage,^35,36^ higher ionic conductivity and lithium transport number, and higher onset exothermic temperature, meaning improved battery safety.^35,37,38^ On graphite electrodes quite different SEI layers were found in LiPF_6_ electrolytes and LiFSI electrolytes.^35^ In the former, the main constituents are solvent reduction products whilst in the latter case salt reduction products are dominating, resulting in a thinner SEI with higher concentration of inorganic species. The main limitation for the LiFSI salt is the poor passivation of the aluminum current collector. However, there are reports of full cells with LiFSI electrolyte where the electrochemical performance is as high as in LiPF_6_ electrolyte, given that FEC is added or charging voltage is limited.^37,39,40^ And if salt purity is high, the Al current collector can remain stable up to 5.0 V in LiFSI electrolyte.^38^

Previous studies with LiFSI based electrolytes have been conducted with electrodes fabricated from nano-silicon materials, of which the native oxide layer constitutes a high fraction of the active material. Significant benefits have been demonstrated by the use of a non-hydrolyzing electrolyte. Micron-sized silicon powders is a different class of promising silicon materials, with advantages such as low cost and low surface area, thus limiting the sites for side-reactions.^41^ In view of the lower fraction of native oxide for these materials, the benefits of non-hydrolyzing electrolytes are less obvious. Furthermore, for micron-sized silicon, the SEI plays a more important role with respect to changes in morphology of the electrode, an inevitable consequence of the repeated expansion and contraction of the silicon during cycling.^42^ In previous studies of nano-silicon electrodes, the composition of the SEI formed in electrolytes composed of LiFSI salt and carbonate solvents has been determined by surface analysis. Little is known about how SEI components are distributed locally inside the porous electrode and on the various surfaces, and there are no simple correlations between SEI composition and properties. Micron-sized silicon is well-suited for studies of local distribution of SEI components, as well as changes in electrode morphology during cycling.

In this study we have therefore systematically characterized micron-sized silicon electrodes in electrolytes composed of 1 M LiPF_6_ and 1 M LiFSI in a carbonate solvent mixture of ethylene carbonate (EC), propylene carbonate (PC) and dimethyl carbonate (DMC), with FEC and VC as additives, by use of electrochemical techniques combined with post mortem studies. The electrodes have been characterized by a range of complimentary techniques, with particular emphasis on identifying morphological changes of electrodes after cycling (by cross section analysis using focused ion beam (FIB), scanning electron microscopy (SEM) and transmission electron microscopy (TEM, EDS, EELS), and electrochemical impedance spectroscopy), local distribution of SEI components inside the electrodes after cycling (TEM) and composition of the SEI (X-ray photoelectron spectroscopy – XPS). In this manner differences in uniformity and mechanical properties of the SEI layer can be revealed, and correlated to the electrochemical performance for these electrolytes.

## 2 Experimental

### 2.1 Electrochemical characterization

Silicon electrodes were prepared by making a slurry of 60 wt% Si (Silgrain®, e-Si 400, a commercially available battery grade silicon from Elkem), with an average particle size of 3 μm, 10 wt% graphite (KS6L, Imerys), 15 wt% carbon black (C-Nergy C65, Imerys) and 15 wt% Na-CMC binder (Sigma Aldrich *M*_w_ ≈ 90 000). A citric acid potassium hydroxide buffer with pH = 3 was used as solvent. The slurry was cast onto dendritic copper foil and dried at 120 °C under vacuum. The electrodes had a loading of around 0.2 mg gSi^−1^, implying a theoretical area capacity of approximately 0.75 mA h cm^−2^.

CR2016 coin cells (Hohsen) were assembled with the silicon electrodes, as working electrode, circular lithium foil as counter electrode and Whattmann glass fiber (250 μm) as separator. Cell assembly was done in an argon filled glove box (O_2_ < 0.1 ppm, H_2_O < 0.1 ppm). Electrolytes used were 1 M LiFSI (>98%, TCI Chemicals) or 1 M LiPF_6_ (battery grade, Sigma Aldrich) in EC : PC : DMC (1 : 1:3 by wt, anhydrous, Sigma Aldrich) with 1 wt% VC (97%, Sigma Aldrich) and 5 wt% FEC (99%, Sigma Aldrich). The particular electrolyte composition was selected based on the fact that FEC and VC are known to improve the performance of silicon electrodes, while EC was partly replaced by PC for improved viscosity of the electrolyte. The amount of electrolyte used was around 70 μL.

Galvanostatic cycling was performed on a BioLogic BCS 805 battery cycler. The C-rate was defined as 1C = 3600 mA g^−1^ (silicon). Prior to cycling, four formation cycles with fixed discharge (lithiation of silicon) capacities of 500, 1000, 1500 and 2000 mA h g^−1^ were conducted at 0.05C. After the formation cycles, the cells were cycled at 1C within a voltage range of 0.05–1.0 V or 0.12–0.9 V, with a constant voltage step after each charge and discharge lasting until the current decreased to 0.5C. Rate performance was performed at 0.1C, 0.2C, 0.5C, 1C, 2C, 5C and 0.1C. Capacity limited cycling was performed at 0.2C, limited to 1000 mA h g^−1^, or a cut-off potential of 50 mV. The upper cutoff voltage was 1.0 V.

Potentiostatic impedance spectroscopy (PEIS) measurements were performed in three-electrode PAT-cells (EL-CELL, Germany) with Li metal as counter and reference electrode on a Biologic VMP300. PEIS measurements were performed after silicon lithiation to 50 mV on cycle 10, 30, 50, 70 and 100. Before the PEIS measurement, the cells were held at constant potential until the current relaxed to 10% of the set current. The resulting data were fitted to a Randles circuit using the software Zview.

In order to determine the diffusion coefficient, the following equation was used as shown in ref. 43 applicable for the high frequency range:1
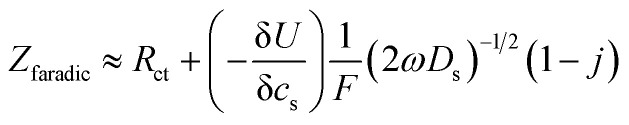
where *U* is the open circuit voltage and *c*_s_ is the concentration of lithium in the solid particles. 
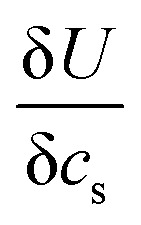
 is found from the linear part of the voltage curve, close to end of lithiation for the given cycle. *D*_s_ is the solid state diffusion coefficient. The capacitance, *C*, is determined from *Z*_Im_ at high frequencies, assuming that faradaic reactions can be neglected, such that 
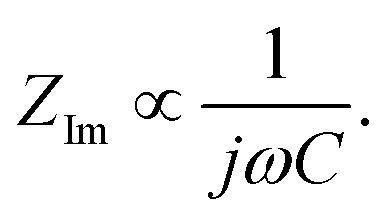


### 2.2 Post mortem characterization

Cross-section samples, as well as cross-section TEM samples, were prepared with a Helios G4 UX focused ion beam (FIB) from FEI. Carbon or Pt layers were first deposited on top of the electrode to protect the area of interest below. The first part of the protection layer was deposited by electron beam assisted deposition. Thick lamellas were cut out and transferred to dedicated Cu TEM half-grids by standard lift-out technique. Coarse thinning was performed with 30 kV ion-beam acceleration voltage. Final thinning was done at 5 and 2 kV on either side of the lamellas to minimize ion-beam induced surface damage. TEM was performed with a double Cs aberration corrected cold-FEG JEOL ARM 200CF, operated at 200 kV. The instrument is equipped with a 100 mm^2^ (0.98 sr solid angle) Centurio SDD for energy dispersive X-ray spectroscopy (EDS) and a Quantum ER GIF for dual electron energy loss spectroscopy (EELS). All spectroscopy was done in STEM-mode and by performing EDS and dual-EELS simultaneously during mapping. Identification of the difference between amorphous and crystalline materials was based on the contrast from the material in normal bright field TEM images when a (small) objective aperture was used. This is possible, as amorphous materials do not give any diffraction contrast, while crystalline materials do. It should be noted that exposure to air could not be avoided during transfer of the samples for TEM and FIB-SEM analysis.

In house XPS characterization was performed by a Kratos Analytical Axis Ultra DLD XPS. The XPS uses an aluminium monochromatic X-ray source operating at 100 W. For each sample, three survey scans with pass energy 160 eV and resolution 0.5 eV from 1200–0 eV were performed in order to identify the elements present on the anode. Next, each core peak of interest underwent 3–7 narrow scans, depending on ease of detection, at pass energy 20 eV with resolution 0.1 eV or 0.05 eV in order to achieve high resolution data. The measurements were done at 10^−9^ torr, with an acceleration voltage of 12 kV and a 12 mA beam current. Sample preparation was carried out in an argon filled glove box, to avoid exposure to ambient atmosphere. An inert transfer arm was used to ensure that the samples were not exposed to air during the transfer from the glove box to the instrument. Data was processed using the software CasaXPS. The adventitious carbon peak in the C 1s spectrum at 285 eV was used for the energy calibration. A tougard background was used when fitting the peaks.

## 3 Results and discussion

### 3.1 Electrochemical performance

**Fig. 1:**
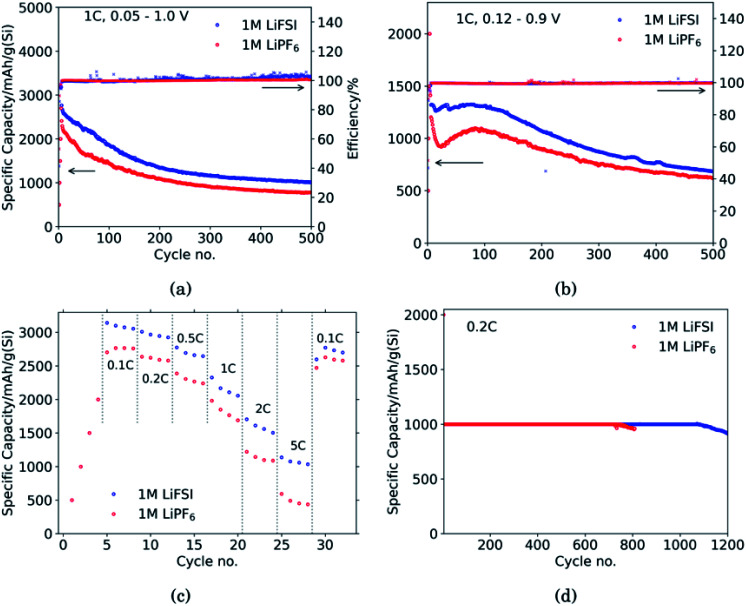
Galvanostatic cycling tests comparing half cells with 60 wt% Si electrodes cycled in 1 M LiFSI (blue) or 1 M LiPF_6_ (red). (a) Galvanostatic cycling in voltage range 0.05–1.0 V, (b) galvanostatic cycling in voltage range 0.12–0.9 V, (c) rate capability in voltage range 0.05–1.0 V, (d) galvanostatic cycling at 0.2C with lithiation of silicon limited to 1000 mA h g^−1^ and delithiation to 1.0 V.

Fig. 1 shows the performance of half-cells with the 60 wt% Si electrodes cycled against lithium metal in electrolytes containing 1 M LiFSI (blue curves) or 1 M LiPF_6_ (red curves).

Cycling in the voltage range 0.05–1.0 V, Fig. 1a, clearly shows higher overall capacity over 500 cycles for the cell with LiFSI electrolyte. It should also be noted that the capacity fade during the first 100 cycles is larger for the electrode cycled in LiPF_6_ than in LiFSI. After 500 cycles, the LiFSI electrode still delivered a capacity above 1000 mA h g^−1^, while the LiPF_6_ electrode was at 775 mA h g^−1^. This translates to the capacity retention after 500 cycles being 36% for the LiFSI electrode and 32% for the LiPF_6_ electrode. Regarding the initial capacity decay, the retention was only 66% for the LiFSI electrode and 59% for the LiPF_6_ electrode after 100 cycles. It should be noted that in Fig. 1a–c, the initial 4 points are the discharge capacities corresponding to the four formation cycles (see Experimental section). As these were fixed to 500, 1000, 1500 and 2000 mA h g^−1^, respectively, the points are overlapping for the two electrolytes. The coulombic efficiencies are above 99.5% for all cycles. The corresponding voltage profiles are provided in the ESI Fig. S1a and b.†

Cycling in the voltage range 0.12–0.9 V, Fig. 1b, results in lower capacity, but improved stability, as expected. Capacity retention of the Si electrodes is better in this voltage range compared to in 0.05–1.0 V, and the coulombic efficiencies are similar. The corresponding voltage profiles are given in the ESI Fig. S1c and d.† The reversible capacity during the first 1C cycle was 1316 mA h g^−1^ for the LiFSI electrode and 1199 mA h g^−1^ for the LiPF_6_ electrode. After 100 cycles the capacity retention for the LiFSI electrode was 98% of it's initial value, while for the LiPF_6_ electrode the retention was 89% of it's initial capacity. After 500 cycles the retention was 52% for the LiFSI electrode and 51% for the LiPF_6_ electrode. The electrode cycling in LiPF_6_ electrolyte show a major drop in capacity during the first 20 cycles before recovering. The recovery effect observed in the LiPF_6_ electrolyte has been observed previously for this material.^42^ In ref. 42, electrodes made from the same silicon material were studied, the only difference was the binder, as Na-CMC was replaced by polyacrylic acid (PAA), but the preparation of the slurry was similar. The electrolyte was composed of LiPF_6_ and the same solvent mixture as used in this work. The fact that the discharge capacity rapidly decline during initial cycles and increased again after cycle 15, was referred to as a pseudo self-healing effect, found to depend on both the binder (even stronger for CMC binders) and the addition of FEC. According to ref. 42, the initial decline in capacity is related to the elasticity of the binder combined with a rather dense SEI limiting the lithiation. The pseudo self-healing is related to the break-up of the SEI, allowing for restoration of the capacity of the silicon electrode. As seen in Fig. 1b, the effect is less significant for the 1 M LiFSI electrolyte, clearly indicating significant differences in the nature of the SEI, which will be elaborated in the next sections.

In the rate capability test, Fig. 1c, the electrode cycled in LiFSI performs better than the electrode cycled in LiPF_6_ at all current rates applied, but the capacity retention when the current is changed back to 0.1C is better for the LiPF_6_ electrode; *i.e.* 85% for the LiFSI electrode and 90% for the LiPF_6_ electrode. The higher capacity retention is most likely a consequence of the lower initial capacity for the LiPF_6_ electrode. In comparison, both the initial capacity and especially the capacity at high rates, is higher for the LiFSI electrode. Hence, the equivalent cycle number for the LiFSI electrode is higher. Also, the higher lithiation degrees at high currents would result in fast volume changes and likely expose this electrode to higher stresses. The difference in capacity obtained with the two electrolytes even at low rates, while at the same time the rate performance of the LiFSI electrolyte is better, indicate that it is not related to differences in conductivity of electrolyte, or the electrode resistance of the counter electrode, but rather to differences in the lithiation, caused by the SEI formed. This will be discussed later.

In the capacity limited cycling, Fig. 1d the electrodes were lithiated up to 1000 mA h g^−1^ for 729 cycles in LiPF_6_ and for 1072 cycles in LiFSI. It should be noted, however, that such half-cell results are relevant for comparison of the two electrolytes, and not for a true assessment of the stability of these electrodes.

A comparison of the first cycle voltage profile obtained in the two different electrolytes is provided in the ESI Fig. S3a.† The SEI forming process is known to differ in these two electrolytes, with an initial dominance of salt reduction for the LiFSI, and a transition to more solvent reduction at lower potentials, while the SEI formation in LiPF_6_ is initiated at lower potentials, and dominated by solvent reduction.^35^ Kang *et al*.^36^ also showed initial reduction at higher potentials in LiFSI electrolyte compared to in LiPF_6_ electrolyte with FEC as additive, though with a larger difference in initial reduction voltage. Cyclic voltammograms have been recorded with an almost identical electrolyte (LiFSI in 1 : 2 EC : DMC with 5 wt% FEC, 1 wt% VC),^44^ showing clearly the presence of reduction peaks at around 1.8, 1.1, 0.75 and 0.55 V. The peak at 1.8 V is related to the reduction of the LiFSI salts,^45^ whereas the reduction peaks at lower potentials are related to reduction of the solvent components.

As the SEI forming reactions are different for the two salts, the kinetics of the reactions will also be different. Therefore the shift in the lithiation potential, assuming that the initial lithiation will start around 0.34 V for crystalline Si, is most likely related to shifts in the share of current for the SEI formation and the lithiation process. The coulombic efficiency is slightly higher for the LiPF_6_ electrolyte during the initial 3 cycles, as can be seen in ESI Fig. S1b,† but already in the 4th cycle the efficiencies are similar. The electrochemical results obtained here for micron sized silicon are similar to what others have found when comparing the same salts for nano silicon electrodes.^34^

### 3.2 Electrode morphology changes during cycling

#### 3.2.1 Cross-sectional analysis

**Fig. 2:**
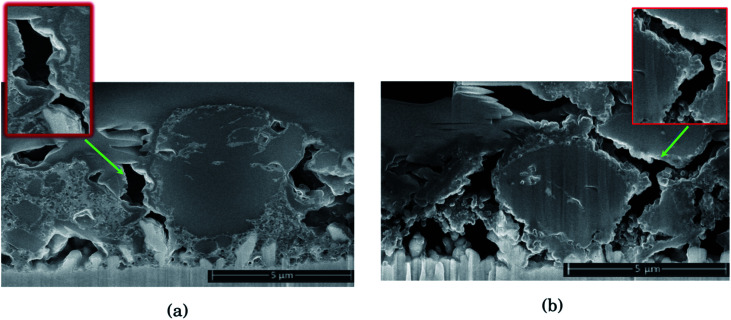
Comparison of cross sections in Si electrodes after 10 cycles in (a) 1 M LiFSI and (b) 1 M LiPF_6_. A layer of carbon has been coated on the electrode surface to protect the electrode structure beneath from the ion beam during sputtering.

Cross-sectional SEM micrographs of the Si electrodes after 10 cycles in LiFSI and LiPF_6_ respectively, are show in Fig. 2a and b. The smooth layer on top of the cross sections is a carbon layer deposited to protect the underlying material during etching with the ion beam. Cross sections of electrodes cycled once and 50 times were also examined, and can be seen in ESI Fig. S3a–d.† Additional close-ups of Fig. 2 is provided in Fig. S3e and f.†

As seen from Fig. 2, for the electrode cycled in LiPF_6_, the voids between the particles resemble cracks in the network of SEI, binder and carbon black, formed as a result of the silicon particles' expansion and contraction. This is especially evident around the large particles on the right side of the cross section seen in Fig. 2b, which is highlighted. The SEI-binder-carbon black network appear to have formed a continuous film across the surface of the particles in the expanded state, and from the roughened surfaces towards the voids between them in the contracted state, the SEI-binder-carbon black network seems to have cracked during the contraction. The cracks indicate a less flexible SEI for this electrolyte, which is more sensitive towards large volume changes.

The large particle on the right side of the cross section in Fig. 2a appear to have cracks going from the surface and inwards which have been filled with SEI products. This could be a result of preferred lithiation paths along particle defects which has become a pore in the particle, and could be the same kind of SEI pore filling as seen in the work by Paul *et al.*^46^ There is less sign of surface roughening after cycling in LiFSI, Fig. 2a.

The SEI-binder-carbon black network for the LiFSI electrode after 50 cycles is more confined around the Si particle (ESI Fig. S3†), and not filling the voids between the particles to the same degree as after 10 cycles. The cross section of the LiFSI electrode after 50 cycles has quite high porosity, and the particles appear less connected compared to after 10 cycles. In the cross section obtained for the LiPF_6_ electrode after 50 cycles on the other hand, pores appear to be clogged by SEI products (see Fig. S3†). For comparison, a cross-sections of a pristine Si electrode are provided in Fig. S4.†

### 3.3 Silicon particle morphology

**Fig. 3:**
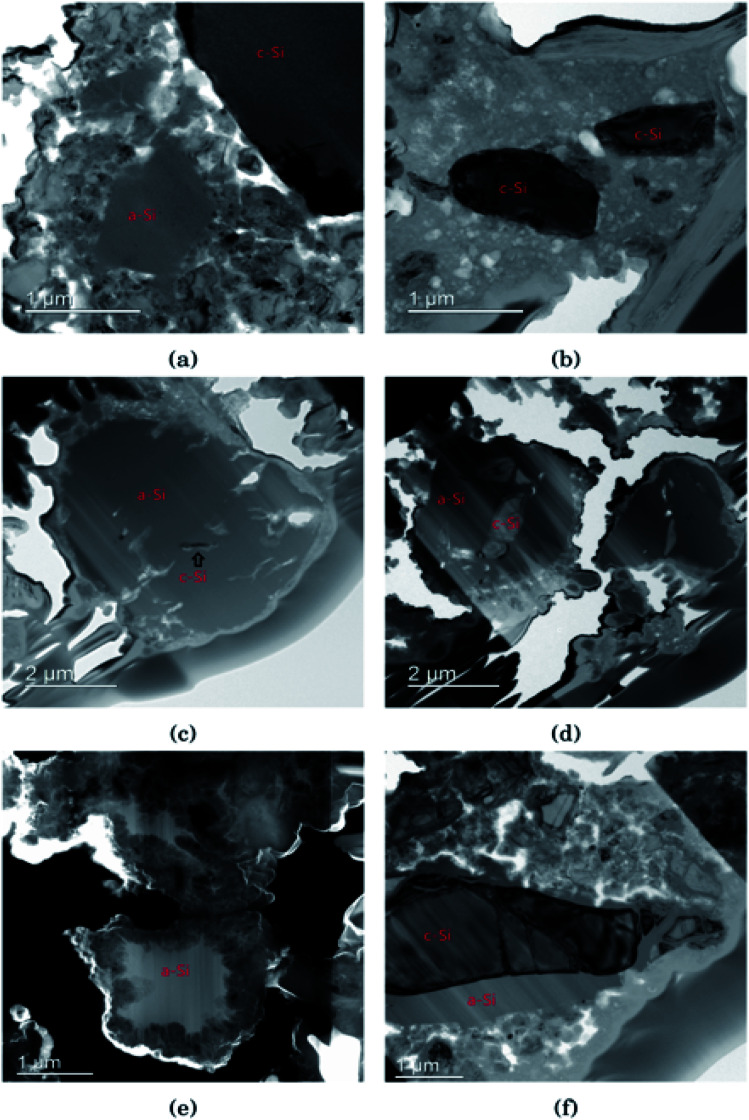
Bright field TEM images after the first cycle in (a) LiFSI and (b) LiPF_6_ (c) after 10 cycles in LiFSI and (d) LiPF_6_ (e) high angle annular dark field scanning transmission electron microscopy image of LiFSI after 50 cycles and (f) bright field TEM image of LiPF_6_ after 50 cycles.

In Fig. 3 the morphology of the Si particles after cycling is shown. Fig. 3a shows a part of the cross section of a Si electrode after the first full cycle in LiFSI where two small amorphous silicon and parts of one large crystalline silicon particle can be observed. Fig. 3b is a micrograph taken after the first cycle in LiPF_6_ where two small crystalline particles can be observed. These particles are about the same size as the largest amorphous particle in Fig. 3a. Fig. 3c shows a Si particle after 10 cycles in LiFSI, where a small crystalline area in the middle of the large Si particle can be seen. Fig. 3d is a micrograph taken after 10 cycles in LiPF_6_, where the Si particles are smaller than in Fig. 3c but quite large areas in the middle of the particles are still crystalline. Fig. 3e is a micrograph taken after 50 cycles in LiFSI, where the structure of the silicon particles have completely changed. A tread-like structure can be observed where the particle edges used to be. The same was seen in the work by Wetjen *et al.*,^47^ who suggested that this roughening of the Si surface and void formation was the result of the dealloying process during delithiation, which can be viewed as a corrosion process.^48^ An additional image illustrating this phenomenon is provided in the ESI Fig. S6.† No such feature can be seen for the LiPF_6_ electrodes. Fig. 3f shows silicon particles after 50 cycles in LiPF_6_, where the larger particles still have large crystalline areas, even close to the particle surface, where the diffusion length for the Li^+^-ions is very short. Fig. 3f also clearly illustrates preferred lithiation paths through the crystalline silicon.

Since more amorphous silicon is observed in the images of electrodes cycled in LiFSI compared to in the images of electrodes cycled in LiPF_6_, it appears that in LiFSI electrolyte, the silicon particles are more or less fully lithiated during the first full cycle. This results in a large expansion of the Si particles, and when the large particles are delithiated they will crack. This cracking is clearly visible in Fig. 3c, after 10 cycles in LiFSI. In the LiPF_6_ electrolyte, large parts of the silicon remain crystalline, meaning this silicon will remain passive in the electrode and not take part in the lithiation/delithiation. The difference in degree of silicon lithiation in the two electrolytes is consistent with the difference in capacity during cycling.

### 3.4 Impedance spectroscopy

**Fig. 4:**
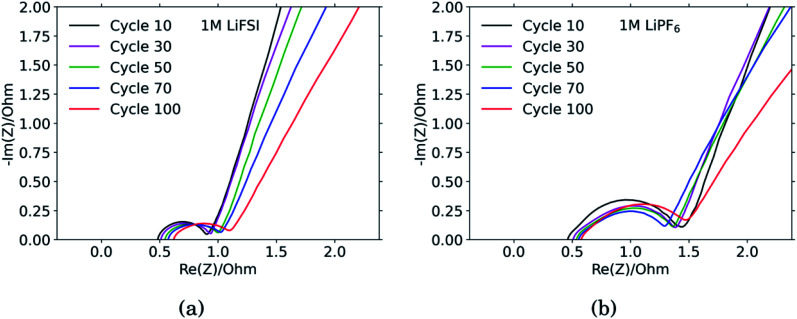
Impedance spectra of Si electrodes cycled in (a) 1 M LiFSI and (b) 1 M LiPF_6_.

The results from the PEIS measurements are summarized in the spectra shown in Fig. 4 and Table 1. The measurements showed very similar electrolyte resistance for the electrodes cycled in LiFSI and LiPF_6_, however the charge transfer resistance was significantly higher for the electrode cycled in LiPF_6_. The lower charge transfer resistance agrees well with the improved rate performance observed for the electrodes cycled in LiFSI compared to in LiPF_6_. In the spectra obtained for the LiFSI electrode, Fig. 4a, a small increase in the charge transfer resistance when going from cycle 30 to cycle 50 can be seen. As seen from the cross section micrographs in ESI Fig. S2,† after 50 cycles in LiFSI, the silicon particles appear to be encapsulated in thick SEI, and also less interconnected than after 10 cycles. The growing thickness of the SEI around the Si particles will increase the interfacial resistance/polarization, due to the limited conductivity of Li^+^ through the SEI. Furthermore, since the SEI is electrically insulating, a thicker SEI will weaken the electrical contact between the Si particles, Si and carbon particles, as well as the contact to the current collector.^1^

**Table 1:** Table of equivalent circuit elements, *i.e.* charge transfer resistance, capacitance and diffusion coefficient from fitting of impedance data for Si electrodes cycled in 1 M LiFSI or 1 M LiPF_6_ after discharge no 10, 30, 50, 70 and 100

Cycle no.	1 M LiFSI			1 M LiPF_6_		
	*R*_ct_ [Ohm]	*C* [F]	*D* [cm^2^ s^−1^]	*R*_ct_ [Ohm]	*C* [F]	*D* [cm^2^ s^−1^]
10	0.46	0.000200	1.53 × 10^−11^	1.0	0.000380	5.71 × 10^−13^
30	0.47	0.000180	1.03 × 10^−12^	0.91	0.000566	5.37 × 10^−13^
50	0.52	0.000190	6.74 × 10^−11^	0.85	0.000736	5.33 × 10^−13^
70	0.51	0.000196	9.93 × 10^−13^	0.74	0.000685	9.43 × 10^−13^
100	0.52	0.000420	1.02 × 10^−10^	0.88	0.000505	8.26 × 10^−15^

The capacitance is a measure of the active area, *i.e.* the surface area accessible by the electrolyte, and thus available for electrochemical reactions. The capacitance is significantly higher for the electrode cycled in the LiPF_6_ electrolyte compared to the electrode cycled in LiFSI. This is consistent with the observed rougher surface (Fig. 2), possibly due to a more uneven SEI layer. The lower capacitance of the LiFSI electrode can similarly be explained by a more even and smooth SEI layer, as would be expected by a layer rich in polymers. The active area, and thus the capacitance, will increase upon expansion of particles and roughening of the surface, and decrease upon clogging of pores, for example. As seen from the results in Table 1, the capacitance of the electrode cycled in LiPF_6_ goes through a maximum over the cycles, while the capacitance of the electrode cycled in LiFSI is stable, and increases in cycle no. 100. The results from the impedance spectra comply well with the post mortem cross-sectional analysis, illustrating the roughened surfaces due to cracking and eventually pore-clogging for electrodes cycled in the LiPF_6_ electrolyte, while the integrity of electrodes from the LiFSI electrolyte is clearly much better preserved.

The diffusion coefficients reported in the table are evaluated from the high frequency region of the impedance spectra, assuming spherical and uniform particles, eqn (1). These values can not be expected to represent highly reliable values. However, it should be noted that the difference between the two electrodes is one to two orders of magnitude, which complies with the observed better utilization of the electrode with the LiFSI electrolyte.

### 3.5 SEI composition

#### 3.5.1 TEM element mapping

**Fig. 5:**
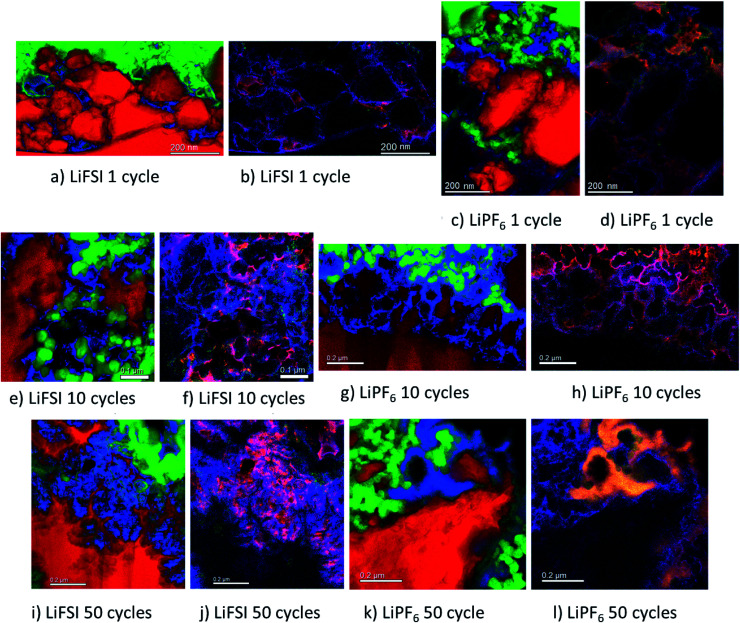
Combined and coloured element maps acquired with STEM-EDS-EELS of Si electrode cycled in 1 M LiFSI once (a and b), 10 times (e and f) and 50 times (i and j), and in 1 M LiPF_6_ once (c and d), 10 times (g and h) and 50 times (k and l). For (a, c, e, g, i and k) red is silicon, green is carbon and blue is lithium. For (b, d, f, h, j and l) red is lithium, green is fluorine and blue is oxygen. The Li, C, O and Si signals/maps were extracted from the EELS data, while the F map was extracted from the EDS signal.

Fig. 5 shows element maps acquired with STEM/EDS/EELS of areas in electrode cross section close to Si particles for the six electrodes discussed above in Section 3.3. Fig. 5a and b show micrographs of the same area only mapped for different elements for the electrode subjected to the first formation cycle in LiFSI. In Fig. 5a, c, e, g, i and k red is silicon, green is carbon and blue is lithium. In Fig. 5b, d, f, h, j and k red is lithium, green is fluorine and blue is oxygen, hence overlap between lithium and fluorine becomes yellow and overlap between lithium and oxygen becomes purple.

The micrographs shown in Fig. 5a–l show clear differences with respect to the amount and location of lithium present in the different samples. For the electrodes cycled in LiFSI, the Li is distributed evenly, and mostly close to the silicon surface in the mapped area. With more cycles, the amount of lithium increases as seen in Fig. 5a, e and i, and after 50 cycles a high amount of lithium can clearly be seen in between the filigree structure of the silicon particle surface. For all the LiFSI samples, the lithium is concentrated around the silicon particles, while only small amounts are detected in the surrounding carbon matrix. This stands in contrast to the LiPF_6_ samples, where much of the lithium is detected in the carbon matrix, Fig. 5c, g and k. In general, it appears that more lithium is detected for the LiPF_6_ samples compared to the LiFSI samples. Among the LiPF_6_ samples, only the electrode cycled 10 times shows lithium located close to the silicon. All the LiPF_6_ samples, and especially the electrodes cycled once and 50 times, show large amounts of lithium in the carbon matrix instead of close to the Si particles. The two electrodes cycled 10 times show the largest difference in amount of detected lithium between the LiFSI and the LiPF_6_ sample. After 50 cycles in LiPF_6_, a somewhat peculiar feature is observed, as seen from Fig. 5k. The lithium is assembled in one big cluster instead of being distributed more evenly around the Si surface. In the left part of the mapped area there is no lithium detected, while in the right upper part of the mapped area, there is a large cluster of lithium between the large Si particle and the carbon black, which stretches out and surrounds a small Si particle and some carbon black particles. Similar features were not seen in any of the electrodes cycled in LiFSI. Since all electrodes are delithiated samples, detection of high amounts of lithium in the carbon matrix must mean that the degree of lithium trapping after cycling in LiPF_6_ is quite high. It should be noted that also after one cycle in LiPF_6_, assemblies of lithium are observed in the carbon matrix (upper part of mapped area, Fig. 5c). Combined element maps of lithium (red), fluorine (green) and oxygen (blue) shown in Fig. 5b, f and j for LiFSI and Fig. 5d, h and l for LiPF_6_, show that much of the lithium detected on the LiPF_6_ samples is in fact LiF due to the clear overlap between Li and F (yellow). For the LiFSI samples the overlap between Li and O (purple), is dominating. Some overlap between Li and F is also seen for the LiFSI electrodes, but to a much smaller extent than for the LiPF_6_ electrodes. Interestingly, for the electrodes cycled 1 and 10 times in LiPF_6_, there seems to be more LiF in the upper part of the mapped areas where the lithium assemblies were seen in the carbon matrix. From the colour of the SEI closest to the silicon, some LiF also seems to be present there, but the overlap and intensity are higher in the carbon black matrix. The large assembly of lithium observed after 50 cycles in LiPF_6_, is clearly LiF, and in this area there is practically no overlap between Li and O. The electrode cycled 50 times in LiFSI, Fig. 5j, on the other hand, is completely dominated by the overlap between Li and O. There is overlap between Li and F also, but not with the same intensity. The LiF on the sample cycled 10 times in LiFSI is visible as small grains, Fig. 5f. Most of these are located close to a silicon surface. Also for the sample cycled once in LiFSI, the overlap between Li and O dominates, while LiF is found as small grains in between the silicon particles.

For the LiFSI electrodes the overlap between lithium and oxygen could originate from Li_2_O, Li_2_CO_3_ and Li_*x*_SiO_*y*_.^9,10^ It should be noted, however, that compounds like Li_2_O will oxidize and form Li_2_CO_3_ even after short exposures to air, which is the case for our samples. The strong overlap between lithium and oxygen close to silicon for the LiFSI electrodes, complies well with a bilayer model of the SEI, *i.e.* with clear separation between inorganic and organic components.^5,17^ For the LiPF_6_ electrodes, on the other hand, there is less overlap between lithium and oxygen, and instead, a large fraction of the lithium is found in LiF clusters.

From these results it appears that there is more LiF on the LiPF_6_ electrodes compared to the LiFSI electrodes. In previous works, addition of FEC has been verified to improve the stability of silicon electrodes during cycling, and typically this has been attributed to the higher amount of LiF detected in the SEI.^21,49^ There is, however, not a general consensus that LiF alone is responsible for the improvements in performance, and some works have rather emphasized the importance of polymeric SEI compounds for the improved reversibility observed upon addition of FEC.^26,32^ In other studies, inorganic components, such as LiOH and Li_2_O, have been suggested to be more important in the SEI because they improve lithium ion conductivity in the SEI layer.^34,50,51^

During spectroscopy mapping in STEM mode, where the dwell time in each pixel is much longer compared to acquisition of a regular STEM image, beam damage by etching of SEI layer was observed, see ESI Fig. S5.† Organic SEI is known to be unstable under the electron beam and can burn off during mapping. Inorganic SEI is much more stable and is less likely to suffer from damage by the electron beam. Fig. S5 in ESI† shows two micrographs of the same area in a cross section of an electrode cycled 10 times in 1 M LiFSI electrolyte, before and after mapping with TEM. As seen from Fig. S5,† after mapping much of the material that originally was found between the two silicon particles, *i.e.* the SEI-binder-carbon black network, has been etched away. Material etching was observed during mapping of the electrodes cycled 10 and 50 times in either electrolyte. For the electrodes lithiated to 500 mA h g^−1^, etching was not observed. After 50 cycles the electrodes cycled in LiFSI and LiPF_6_ showed similar degree of etching during mapping. After 10 cycles however, the electrode cycled in LiFSI clearly suffered far more material etching than the electrode cycled in LiPF_6_. For the LiFSI electrode, the etching was more severe further away from the silicon particles, *i.e.* the outer layers of the SEI. Overall, the etching caused by the electron beam indicate that there are more organic components in the SEI formed in LiFSI electrolyte after 10 cycles than in the SEI from LiPF_6_, especially at some distance from the silicon. Also, the inorganic species close to the Si surface and the organic components further away are clearly separated.

Based on the colour intensity in the TEM element maps, more oxygen is detected in the electrodes cycled in LiFSI electrolyte compared to in LiPF_6_ electrolyte. For the electrode cycled 50 times in LiFSI, the Si filigrees are covered by oxygen, and also the electrode cycled 10 times in LiFSI has quite a lot of oxygen on the silicon. This stands in contrast to the LiPF_6_ samples where hardly any oxygen is detected on the silicon in the maps. The oxygen detection suggests that there are more oxygen containing, inorganic species on the LiFSI electrodes compared to the LiPF_6_ electrodes. Philippe *et al.* found that upon cycling in LiFSI electrolyte, the favorable conversion products of Li_2_O and Li_4_SiO_4_ formed upon lithiation are preserved, and thus also the favorable interaction with the binder and the active material.^34^ Due to the formation of HF in LiPF_6_ electrolyte, the Li_2_O is etched away, and SiO_*x*_F_*y*_ is formed.^34^ Hence, LiPF_6_ as electrolyte salt, compared to LiFSI, can result in weaker interaction between silicon and binder, which would increase the probability of SEI cracking during operation, in addition to causing fluorination and dissolution of favorable oxygen containing SEI species.

#### 3.5.2 XPS

Table 2 shows the composition of the SEI detected with XPS for electrodes cycled to 500 mA hg^−1^ (first formation cycle), cycle 10 (delithiated state) and cycle 50 (delithiated state) in either 1 M LiFSI or 1 M LiPF_6_ electrolyte. The elemental composition of a fresh, uncycled Si electrode is also included in the table, where C and O originate from the binder. Silicon is detectable after the 1st lithiation and 10th cycle for the LiFSI electrodes, while only after the first lithiation for the LiPF_6_ electrodes. This indicates that a thinner SEI is formed with LiFSI. From the XPS survey results, Table 2, it was seen that the LiFSI electrodes had higher percentages of O and C than the LiPF_6_ electrodes, which could indicate more organic SEI components, in agreement with the observed etching during TEM mapping. While the elemental composition of the Si electrodes is similar after 10 and 50 cycles in LiFSI, there is an increase in F and Li, and a decrease in C and O for the SEI in LiPF_6_ over these cycles.

**Table 2:** Elemental composition in at% of Si electrode surfaces before and after cycling in either 1 M LiFSI or 1 M LiPF_6_ electrolyte, acquired from survey spectra after XPS measurement

Element	Si	O	C	F	Li	P	N	S
Fresh electrode	5.4%	20.2%	73.7%	—	—	—	—	—
1st lith LiFSI	0.3%	16.3%	28.8%	17.9%	31.0%	—	1.9%	3.9%
10h cyc LiFSI	0.2%	20.2%	43.3%	12.2%	17.3%	—	2.0%	4.0%
50th cyc LiFSI	—	20.2%	44.4%	12.5%	17.0%	—	1.7%	3.1%
1st lith LiPF_6_	0.3%	10.4%	18.8%	23.7%	44.8%	2.0%	—	—
10th cyc LiPF_6_	—	15.7%	32.5%	22.7%	27.5%	1.7%	—	—
50th cyc LiPF_6_	—	12.7%	28.1%	25.1%	32.9%	1.2%	—	—

**Fig. 6:**
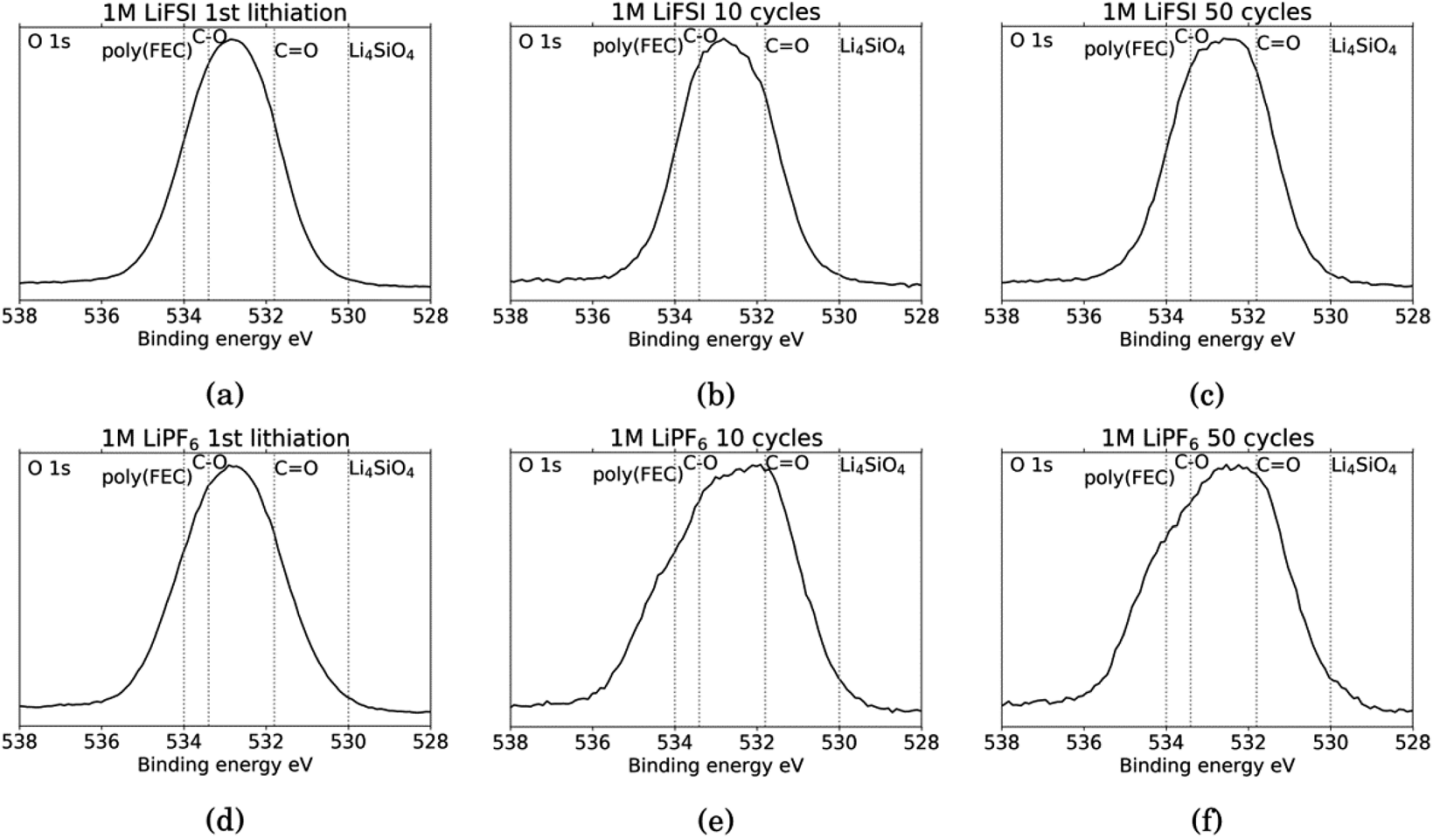
O 1s spectra for Si electrodes cycled to 1st lithiation, cycle 10 and cycle 50 in LiFSI (a–c) respectively, and in LiPF_6_ (d–f) respectively.

Fig. 6 shows the O 1s spectra for all the cycled samples. It is agreed upon in literature that features that can be determined from O 1s spectra is C=O at around 531.8 eV and organic C–O at around 533.4 eV.^18,27,31,52^ Carbonates (Li_2_CO_3_) will contribute only to the C=O feature, while organic components will contribute both to the C–O feature and the C=O feature.^52^ In addition, electrodes cycled in FEC can have an additional shoulder at 534 eV assigned to poly(FEC), which is related to a FEC reduction product.^29^ A feature at around 530 eV can be assigned to lithium silicate (Li_4_SiO_4_).^11^ After the first lithiation, the O 1s spectra for the LiFSI sample and the LiPF_6_ sample, Fig. 6a and b, are very similar. The only difference is a slightly more prominent shoulder at 534 eV, indicating FEC reduction products, for the LiPF_6_ sample. For the LiFSI samples, the spectra are close to identical after the 1st lithiation and the 10th cycle. The peak in the spectra after 10 cycles in LiFSI is centered closer to the binding energy of organic C–O than C=O, while the peak in the spectra after 10 cycles in LiPF_6_ is centered closer to the C=O binding energy. Since higher percentages of oxygen are detected on the LiFSI electrodes compared to the LiPF_6_ electrodes, which cannot be attributed to Li_2_O or Li_2_CO_3_, this indicates that more organic components are detected for the LiFSI electrodes. The LiPF_6_ electrode after 10 cycles has a shoulder at the binding energy of FEC reduction products which the LiFSI sample is lacking. After 50 cycles, the peak in the spectrum for both the LiFSI and the LiPF_6_ sample is centered between the binding energy for C–O and C=O, though slightly closer to C=O than C–O. The shoulder indicating again FEC reduction products is more prominent for the LiPF_6_ electrode after 50 cycles than after 10 cycles.

**Fig. 7:**
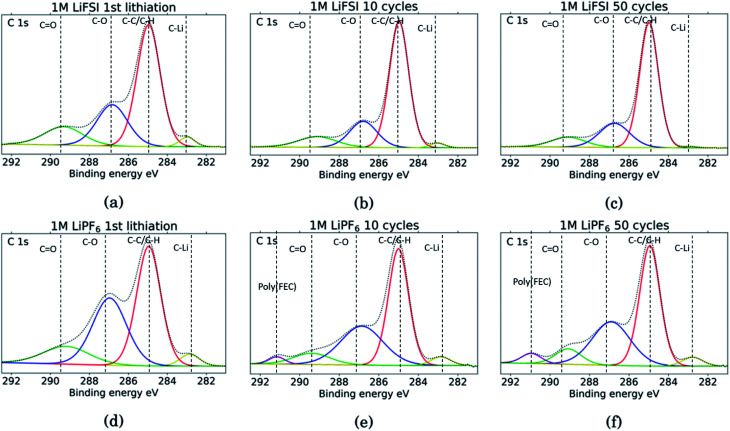
C 1s spectra for Si electrodes cycled to 1st lithiation, cycle 10 and cycle 50 in LiFSI (a–c) respectively, and in LiPF_6_ (d–f) respectively.

The C 1s spectra are shown in Fig. 7. At low binding energies, there is a small peak at 282.8 eV, in yellow, assigned to lithiated carbon.^35,52^ For the LiFSI electrodes, this peak can be seen after the 1st lithiation and after the 10th cycle, while after the 50th cycle the peak is almost gone. The intensity of this peak decrease from the 10th to the 50th cycle, while the atomic% of carbon is unchanged, indicating a growing SEI. All the LiPF_6_ samples show this peak for lithiated carbon with higher intensity than the corresponding LiFSI samples, even if the atomic% of carbon is reduced in the 50th cycle compared to the 10th for the LiPF_6_ electrodes. Also, the fact that the peak increase from cycle 10 to 50 for the LiPF_6_ electrodes must mean that there are more lithium being trapped in carbon during cycling.

The binding energies for the next two peaks are well established in literature, C–C/C–H (adventitious carbon) at 285 eV, in red, and C–O at 286.8 eV, in blue.^10,11,18,20,27,29,31,34,53^ These peaks are found for all the samples, however, the C–O peak in blue is much larger for the LiPF_6_ samples compared to the LiFSI samples, while overall, the atomic% of carbon is lower in the LiPF_6_. In addition, this peak is larger for the sample after lithiation compared to after delithiation for both the LiFSI samples and the LiPF_6_ samples. In general, the C 1s spectra for the LiFSI electrodes show higher intensity of the C–C peak compared to the other peaks in the spectra than in the C 1s spectra for the LiPF_6_ electrodes. This can indicate thinner SEI for the LiFSI electrodes because more carbon black is detected, in line with the at% of silicon shown in Table 2.

The next peak, in green, at 289–290 eV is assigned to a C=O feature (carbonates), which is also agreed upon in literature.^10,11,18,20,27,29,31,34,53^ The C=O peak is quite wide for several of the samples, indicating that it originates from an overlap of features from several compounds, including the binder. It is for example common to fit a peak for CO_2_ at 288.5 eV.^10,11,31,34^ Alternatively, the peak broadening is related to charging effects.^54^ Comparing the three LiFSI electrodes, the green peak for carbonate seems to have a smaller area than the blue peak for organic C–O features, in agreement with the O 1s spectra, as seen in Fig. 6a, c and e.

For the LiPF_6_ electrodes, after the 10th and 50th cycle, there is a feature found at 291 eV, in purple, which is not apparent in the C 1s spectra for the LiFSI electrodes. This is consistent with a –CHF–OCO_2_-type reduction product from FEC.^18,20,27,31^ This indicates that more FEC has reduced on the LiPF_6_ electrodes, possibly due to more cracking of the SEI. It is seen from the voltage profile of the first lithiation, ESI Fig. S1,† that electrolyte reduction starts at a higher potential for the cells with LiFSI than the ones with LiPF_6_. In LiPF_6_ electrolyte, the SEI formation is initiated by solvent reduction, and FEC is the first to reduce, while in LiFSI electrolyte the salt is known to reduce first.^34,35^ The fact that no signature for this FEC reduction product is seen in the C 1s spectra for the LiFSI samples is unexpected, since FEC would reduce before other solvents in the electrolyte. This could indicate that the salt reduction products contribute to passivation of the electrode, and is also consistent with the few cracks observed in the cross-sections.

**Fig. 8:**
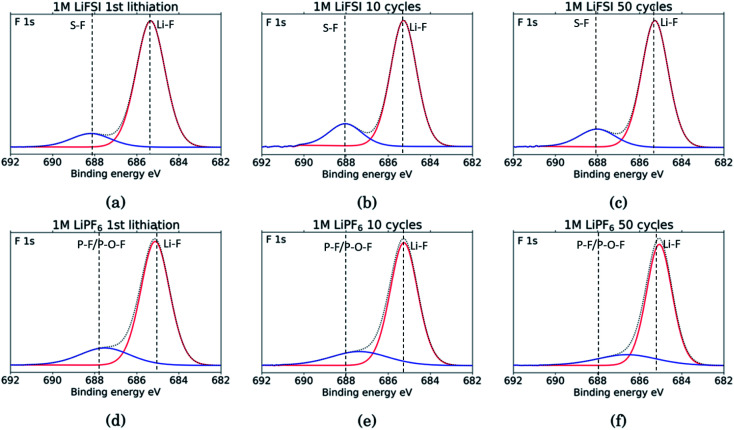
F 1s spectra for Si electrodes cycled to 1st lithiation, cycle 10 and cycle 50 in LiFSI (a–c) respectively, and in LiPF_6_ (d–f) respectively.

The F 1s spectra, Fig. 8, show only two peaks. For all six samples the major peak, in red, is located at 685 eV and is assigned to LiF.^10,11,18,27,29,31,34,53^ For the three LiFSI samples, the second peak in blue at 688 eV is assigned to the LiFSI reduction products.^34^ For the three LiPF_6_ samples the blue peak is assigned to LiPF_6_ reduction products, P–F or P–O–F. An interesting feature for these LiPF_6_ samples is a shift in the blue peak seen from cycle 10 to 50. In the sample cycled 10 times, the blue peak is located at 687.4 eV which correlates well with the LiPF_6_ salt.^10,11,18,29,53^ For the sample cycled 50 times this peak has shifted to 686.6 eV which could indicate contribution from a F–C environment, possibly from reduction of FEC.^18,27^ This result combined with a growing peak at 291 eV in the C 1s spectra, and a shoulder on the peak in the O 1s spectra at 534 eV, supports the assumption of detection of FEC reduction products. Since the blue peaks in the F 1s spectra for the LiPF_6_ samples are quite wide, it is likely that there is some overlap between the peak for LiPF_6_ salt and F–C components in both spectra. This is consistent with the results from the C 1s spectra, and the observed presence of FEC reduction products, most likely related to a less flexible SEI with more cracks.

Comparing the amount of LiF detected by the XPS, results show that the percentage of LiF for the LiPF_6_ samples is twice that of the LiFSI samples after the 10th and 50th cycle, 20% *vs.* 10% for LiPF_6_ and LiFSI, respectively. After the first lithiation the values are more similar, around 24% for the LiPF_6_ sample and 18% for the LiFSI sample. The elemental analysis based on the survey scan, Table 2, showed an increase in the ratio between Li and F from the 10th to the 50th cycle in LiPF_6_ electrolyte, indicating that over the cycles, lithium has been trapped in the SEI in components other than LiF. This is consistent with the established views of the SEI composition, *i.e.* that the outer layers are dominated by organic components. Also, the cross section images suggested more cracks in the SEI for the electrodes cycled in LiPF_6_ compared to the electrodes cycled in LiFSI Fig. 2, which would result in higher consumption of lithium during new SEI formation for the LiPF_6_ electrodes. All together these results support the hypothesis that one of the major differences between these two salts is that cycling in LiPF_6_ results in more irreversible trapping of lithium in the SEI, as LiF, and in lithiated carbon, than cycling in LiFSI does. As discussed previously, high amounts of LiF in the SEI does not seem beneficial for the electrochemical performance, as the electrodes cycled in LiFSI outperforms the electrodes cycled in LiPF_6_.

In the Li 1s spectra, shown in Supplementary Information Fig. S7,† both the LiFSI and the LiPF_6_ samples show a shift in the peak towards lower energies from being centered closer to the binding energy for LiF after the first lithiation to being centered closer to the binding energy for Li_2_CO_3_ after the 50th cycle.

In summary, the improved rate performance and the lower charge transfer resistance observed from the electrochemical impedance spectra, indicate that the SEI formed in LiFSI electrolyte has a higher ionic conductivity than the SEI formed in LiPF_6_. With LiFSI, an inner, inorganic layer is formed due to the reduction of LiFSI at high potentials.^34,35^ We suggest that a major reason for the different features of the SEI from the two salts is the initial SEI formation. The inorganic layer has higher elastic moduli than the organic phase, so if a thin, inorganic layer is allowed to form on the Si surfaces from LiFSI reduction, this SEI can better handle the volume changes. If the inner layer, on the other hand, is not fully formed, so that the SEI becomes a mixture of organic and inorganic phases instead of these being separated in two layers, volume expansion is more likely to result in cracks in the SEI. Also, as was previously concluded, in an LiFSI electrolyte, the native oxide layer on silicon is not fluorinated, which preserves the favorable interactions between the binder and the active material.^34^ In a LiPF_6_ electrolyte on the other hand, the SiO_2_ layer is fluorinated, changing the Si surface and thus the interactions with the binder.

The Li^+^ conductivity is highest along grain boundaries of the inorganic components. A complete, undamaged, inorganic phase, rich in oxygen containing species, at the silicon surface could explain the higher degree of lithiation of Si, lower charge transfer resistance and lower amount of Li trapped in the carbon matrix for the LiFSI electrodes. While lower Li conductivity in the SEI from LiPF_6_ could be due to a more mixed distribution of organic and inorganic components resulting in less grain boundaries between inorganic components, and potentially creating pinning points in the SEI where lithium is trapped. Already after the first formation cycle in LiPF_6_, assemblies of lithium with some fluorine in the carbon matrix are observed. Hence, already from the first cycle the SEI can hamper the Li^+^ conductivity and result in Li trapping and inhomogeneous lithiation, as was also observed by TEM analysis (Fig. 3). Results from the electrochemical performance tests showed that the capacities for the LiPF_6_ electrodes were initially lower, and the decay was steeper, than for the LiFSI electrodes over the first 50 cycles.

For electrodes cycled in LiFSI, there are no signs of cracks in SEI-binder-carbon black network, the SEI composition, the electrode–electrolyte interfacial area is more stable with cycling, and FEC reduction products could not be detected. It is therefore also possible that a lower consumption rate of FEC contributes to the improved stability in this electrolyte as compared to the LiPF_6_ electrolyte.

## 4 Conclusion

In this work the electrochemical performance of electrodes containing 60 wt% of micron sized silicon has been systematically investigated in combination with electrolytes with a mixture of carbonate as solvent (EC : PC : DMC 1 : 1 : 3 with 1 wt% VC and 5 wt% FEC) and LiPF_6_ or LiFSI salt. Improved performance of electrodes investigated together with the LiFSI salt was observed both with respect to capacity, cycling stability, rate performance and number of cycles obtained during limited capacity cycling (>1000 cycles at 1000 mA h g_Si_^−1^) as well as the electrode resistance obtained by electrochemical impedance spectroscopy. Electrode morphological changes, SEI composition and local distribution of SEI components were studied by FIB-SEM, XPS, TEM and electrochemical impedance spectroscopy. The SEI formed with the LiFSI electrolyte is composed of a thin, predominantly inorganic layer, primarily resulting from the reduction of the LiFSI salt at high potentials, and an outer layer dominated by organic components. The interfacial electrode–electrolyte area as determined from impedance spectra, was almost constant for the LiFSI electrolyte during cycling, but went through a maximum for the LiPF_6_ electrolyte. The SEI formed in LiFSI was more homogeneous, flexible and with a lower resistivity compared to the SEI formed in LiPF_6_. For the latter, the composition appeared to be a mixture of organic and inorganic compounds, due to the lower reduction potential of the LiPF_6_ salt. The SEI formed with LiPF_6_ was found to be less homogeneous and less flexible, and more resistive. The high amounts of LiF observed in the SEI for the LiPF_6_ sample does not appear beneficial for the performance, and were observed primarily as clusters formed in the carbon black matrix. The inhomogeneous SEI is also consistent with the incomplete lithiation of Si particles observed by TEM after cycling in LiPF_6_.

## Author contributions

Karina Asheim: conceptualization, data curation, formal analysis, visualization and writing of the original draft. Per Erik Vullum: TEM analysis, supervision and review and editing of the manuscript. Nils P. Wagner: conceptualization, supervision and review and edition of the manuscript. Hanne Flåten Andersen: fabrication of electrodes, review and editing of the manuscript. Jan Petter Mæhlen: conceptualization, funding acquisition, supervision, review and editing of the manuscript. Ann Mari Svensson: Conceptualization, funding acquisition, supervision, review and editing of the manuscript.

## Conflicts of interest

There are no conflicts of interest to declare.

## Supplementary Material

RA-012-D2RA01233B-s001
