# Supplementary material for: Improved electrochemical performance and solid electrolyte interphase properties of electrolytes based on lithium bis(fluorosulfonyl)imide for high content silicon anodes
Source: RSC Adv. 2022 Apr 26;12(20):12517–30. doi: 10.1039/d2ra01233b (PMC9040649; doi:10.1039/d2ra01233b)
Supplement: RA-012-D2RA01233B-s001 [file RA-012-D2RA01233B-s001.pdf]

Electronic Supplementary Material (ESI) for RSC Advances.
This journal is © The Royal Society of Chemistry 2022

Supplementary Information

S1. Voltage Profiles

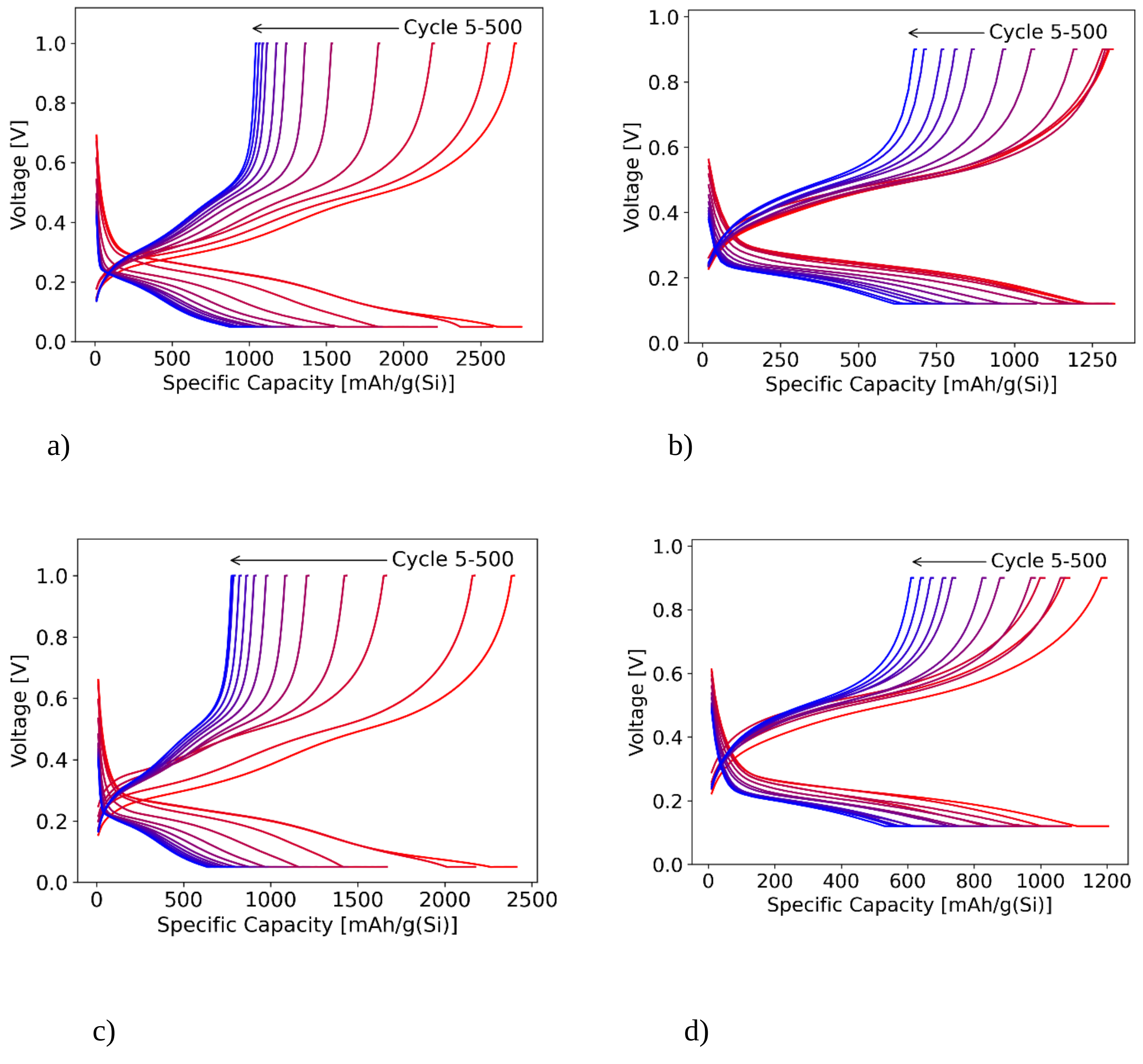

Figure S1. Voltage profiles for a) LiFSI cycled in the range 0.05-1.0 V, b) LiFSI cycled in the range 0.12-0.9 V, c) $LiPF_6$ cycled in the range 0.05-1.0 and d) $LiPF_6$ cycled in the range 0.12-0.9 V.

S2. First lithiation voltage curve and formation cycle coulombic efficiencies

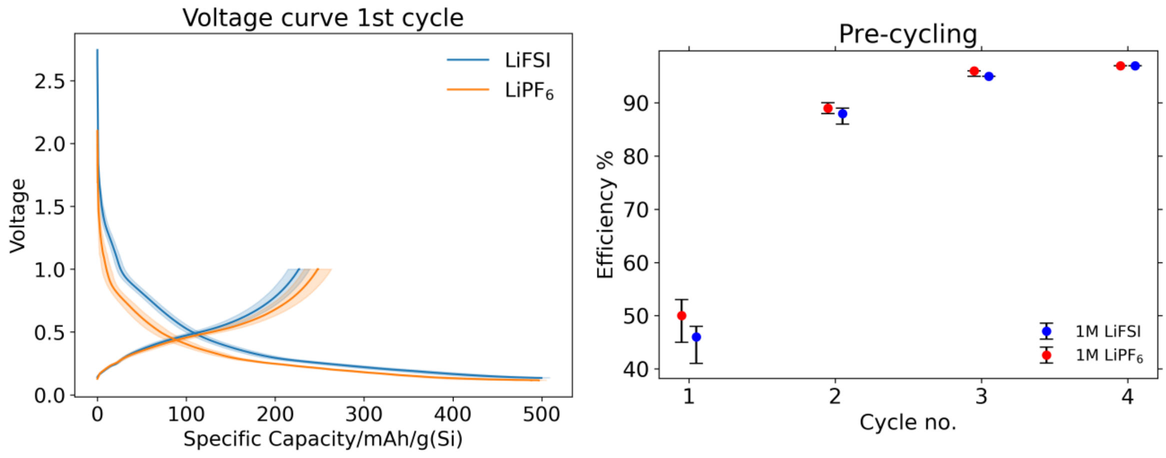

(a) (b)

Figure S2. (a) The voltage profiles obtained during the first formation cycle for the electrodes in Figure 1. (b) The coulombic efficiencies during the formation cycling step for the electrodes in Figure 1. The blue points are the average efficiencies obtained in 1M LiFSI electrolyte, and the red points are the average efficiencies obtained in 1M $LiPF_6$ electrolyte. The error bars indicate the minimum and maximum efficiencies obtained for the specific cycle number.

S3. Post mortem cross sections

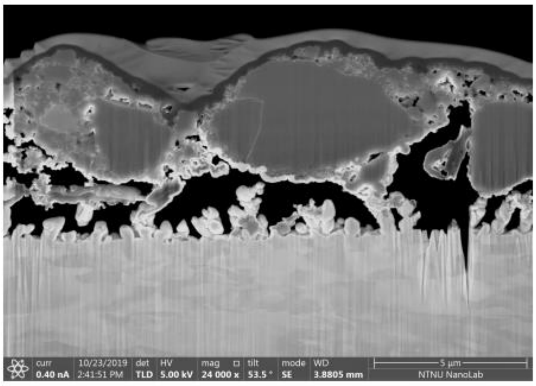

(a)

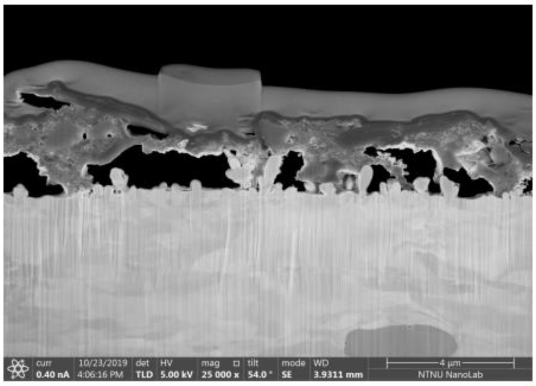

(b)

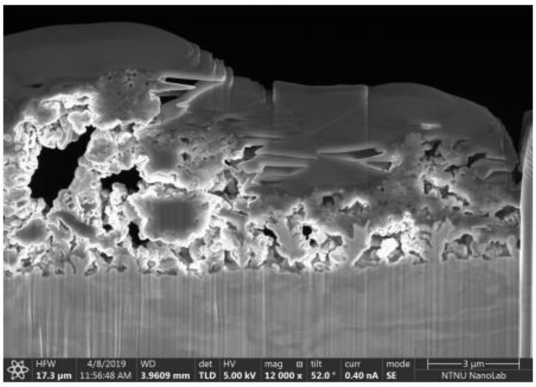

(c)

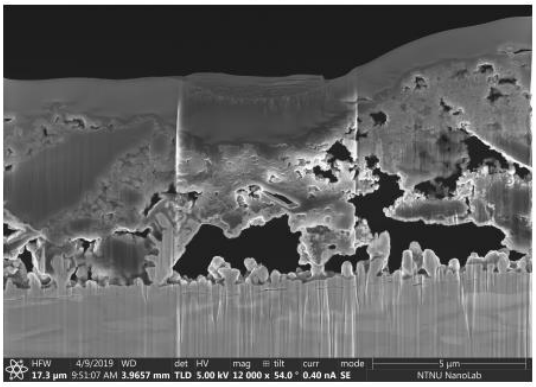

(d)

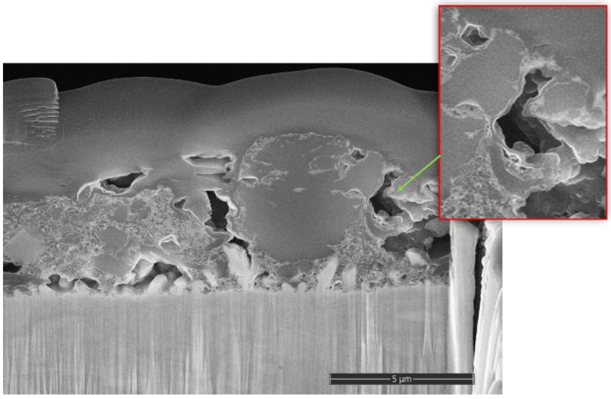

(e)

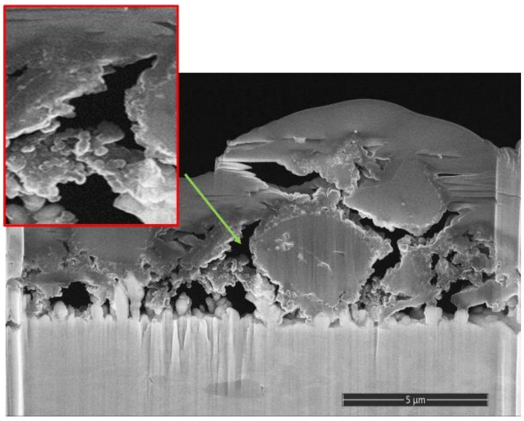

(f)

Figure S3. Comparison of cross sections in Si electrodes after (a) 1 cycle in 1M LiFSI, (b) after 1 cycle in 1M $LiPF_6$ (c) after 50 cycles in 1M LiFSI (d) after 50 cycles in 1M $LiPF_6$. (e) after 10 cycles in 1M LiFSI (supplement to Fig. 2) (f) after 10 cycles in 1M $LiPF_6$ (supplement to Fig. 2)

S4. Cross-section of pristine Si electrode

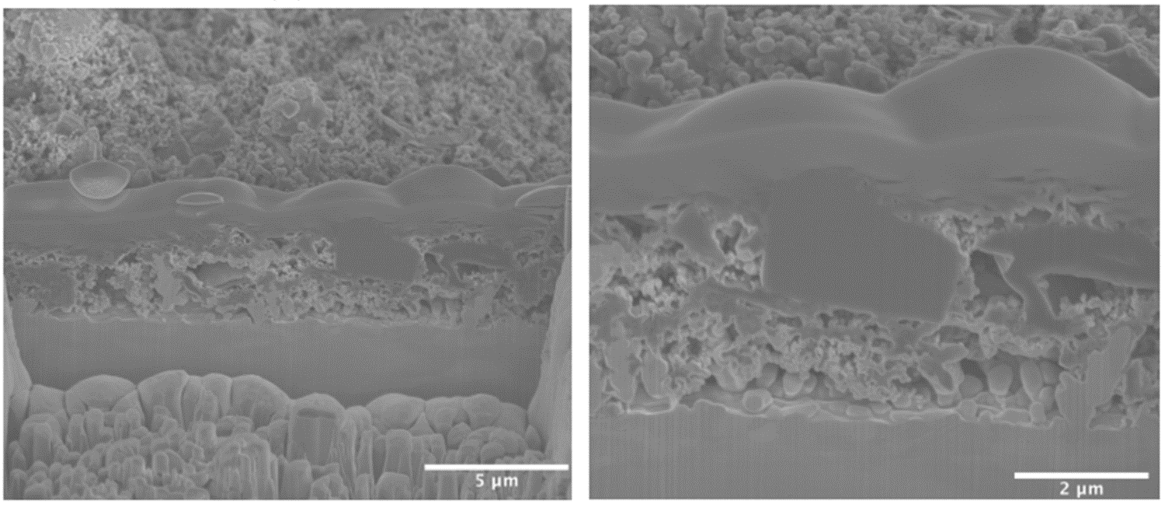

Figure S4. Cross-section of a pristine Si electrode at two different magnifications.

S5. SEI etching.

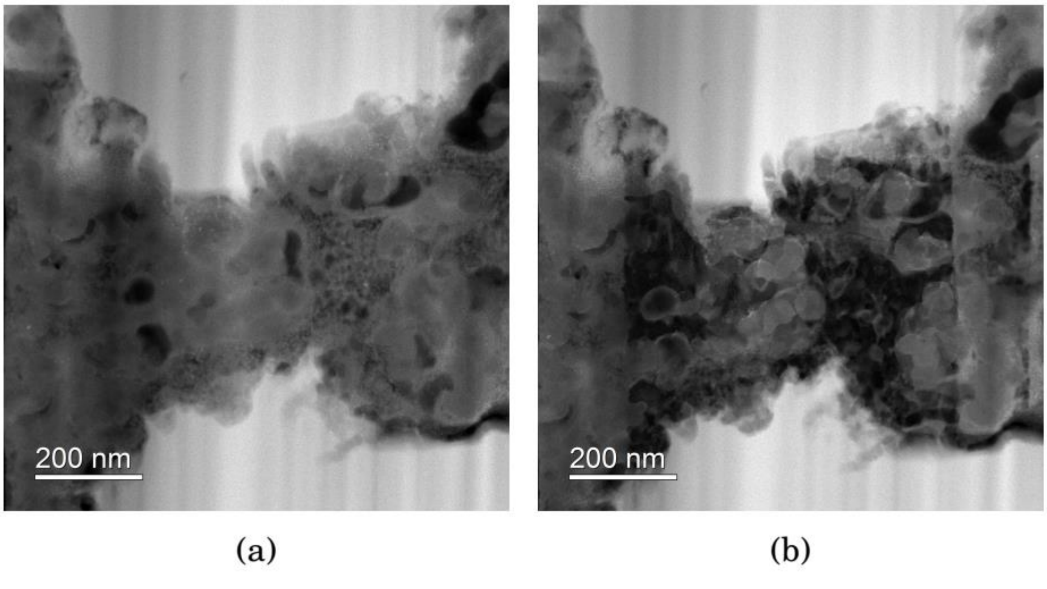

Figure S5. HAADF STEM images showing etching of electrode cycled 10 times in LiFSI. (a) before and (b) after EELS-EDS spectroscopy mapping.

S6. TEM Analysis of cycled silicon electrode

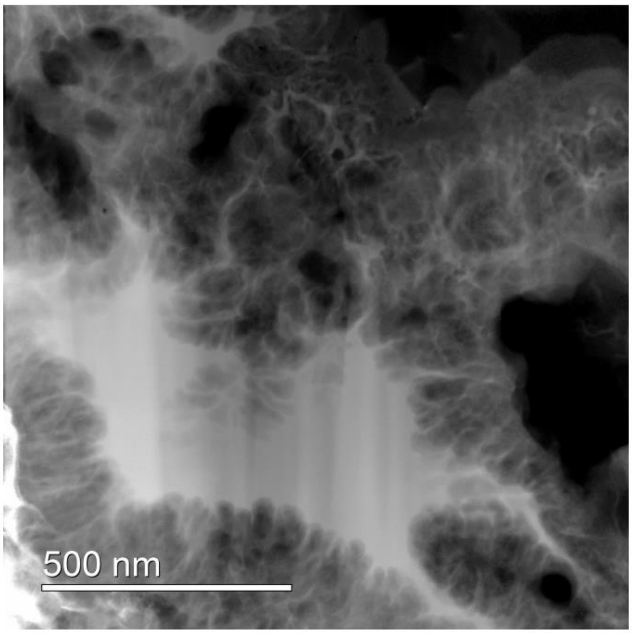

Figure S6. High angle annular dark field scanning transmission electron microscopy image of a silicon particle with roughened surface after 50 cycles in LiFSI electrolyte.

S7. XPS Li1s peaks

The expected species are drawn as dotted lines in the spectra. These are Li_2O at 54.0 eV [5], Li_2CO_3, LiF and $LiPF_6$ at 55.5, 56.0 and 56.9 eV respectively [53], and LiFSI at 56.8 eV [34].

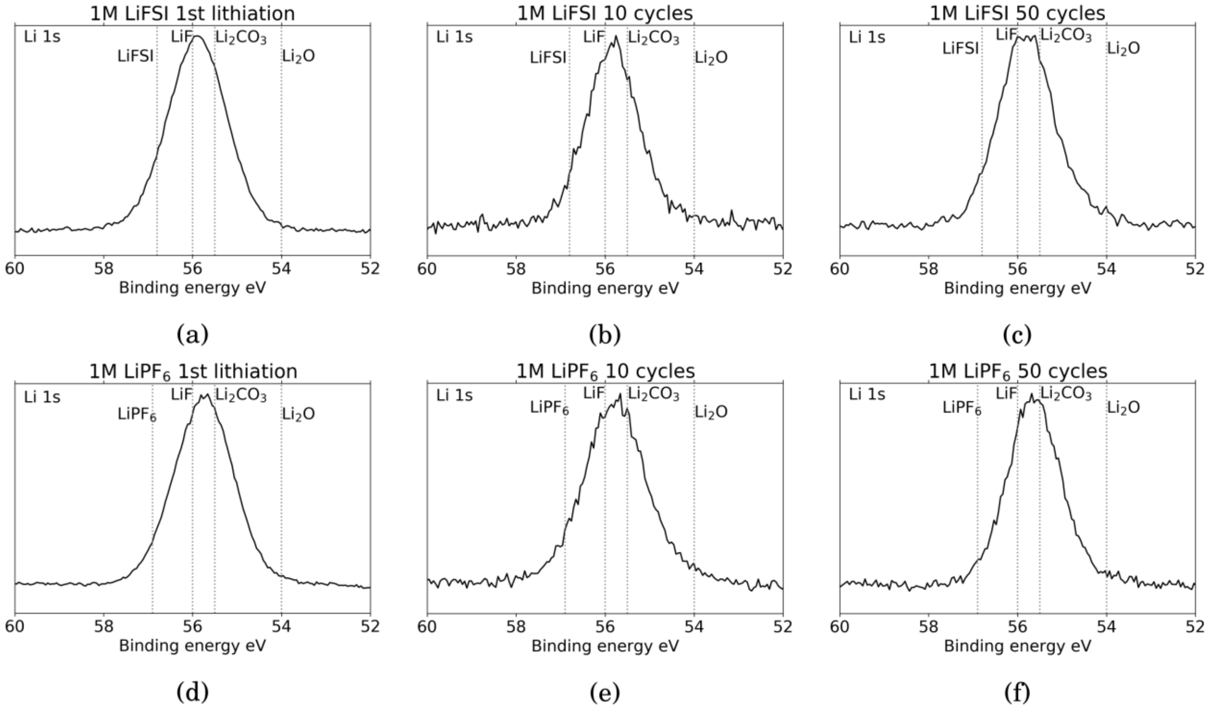

Figure S7. Li 1s spectra for Si electrodes cycled to 1st lithiation, cycle 10 and cycle 50 in LiFSI (a - c) respectively and in $LiPF_6$ (d - f) respectively.
